# Reduced thermal sensitivity and increased opioidergic tone in the TASTPM mouse model of Alzheimer's disease

**DOI:** 10.1097/j.pain.0000000000000644

**Published:** 2016-06-09

**Authors:** Yahyah Aman, Thomas Pitcher, Raffaele Simeoli, Clive Ballard, Marzia Malcangio

**Affiliations:** Wolfson Centre for Age Related Diseases, King's College London, London, United Kingdom

**Keywords:** Alzheimer's disease, Nociceptive threshold, Transgenic mice, Opioids

## Abstract

Individuals with Alzheimer's disease (AD) are in susceptible patient groups in which pain is an important clinical issue that is often underdiagnosed. However, it is unclear whether decreased pain complaints in patients with AD result from elevated pain tolerance or an impaired ability to communicate sensations. Here, we explored if AD-related pathology is present in key regions of the pain pathway and assessed whether nociceptive thresholds to acute noxious stimulation are altered in the double-mutant APPswe × PS1.M146V (TASTPM) transgenic mouse model of AD. TASTPM mice exhibited an age-dependant cognitive deficit at the age of 6 months, but not at 4 months, a deficit that was accompanied by amyloid plaques in the cortex, hippocampus, and thalamus. In the spinal cord, β-amyloid (APP/Aβ) immunoreactivity was observed in dorsal and ventral horn neurons, and the expression of vesicular glutamate transporter 2 (VGLUT2) was significantly reduced, while the expression of the inhibitory peptides enkephalins was increased in TASTPM dorsal horn, consistent with an increased inhibitory tone. TASTPM mice displayed reduced sensitivity to acute noxious heat, which was reversed by naloxone, an opioid antagonist. This study suggests that increased inhibition and decreased excitation in the spinal cord may be responsible for the reduced thermal sensitivity associated with AD-related pathology.

## Abstract 2

Increased inhibition and decreased excitation in the spinal cord may be responsible for the reduced thermal sensitivity in TASTPM transgenic mouse model of Alzheimer disease.

## 1. Introduction

Alzheimer's disease (AD) is a progressive and irreversible age-related neurodegenerative disease. It is the most common form of dementia (>60%) in the elderly population, affecting around 35 million people worldwide and ∼800,000 people in the United Kingdom.^^25,31,43^^ The prevalence of AD is approximately 5% in people older than 65 years, increasing to approximately 30% by the age of 85 years.^^22^^ The aetiology of the disease is complex due to the heterogeneity of the disorder. Alzheimer's disease is clinically characterised by a global deficit in cognition ranging from loss of memory to impaired judgement and reasoning.^^43^^ Neuropathological hallmarks of AD are extracellular β-amyloid (Aβ) plaques and intracellular neurofibrillary tangles composed of abnormally hyperphosphorylated microtubule-associated protein tau. These hallmarks are accompanied by neuronal loss, synaptic dysfunction, brain atrophy, and inflammation.^^40^^

Accumulating evidence indicates that a significant alteration in pain perception is an important clinical issue in patients with AD. Assessment and treatment of pain in AD are often difficult, thereby having a negative impact on the quality of life.^^1,8^^ In fact, prevalence of pain in dementia has been estimated to be ∼50-80%, and the presence of pain is associated with distress and neuropsychiatric symptoms.^^17,8^^ Recent studies suggest that patients with AD do not report pain as often and are prescribed analgesics less frequently compared with age-matched individuals without AD.^^36,7^^ In addition, patients with AD manifest increased tolerance to ischemic pain but no change in the sensory discriminative aspect of pain compared with healthy controls, thereby suggesting an alteration in the emotional dimension of pain.^^2^^ Moreover, histological evidence shows AD-related cytoskeletal pathology and Aβ deposition in the intralaminar nuclei of the thalamus and neurofibrillary tangle in the spinal cord.^^38,34^^ Both the spinal cord and the thalamus are key regions in the pain pathway which are involved in processing and relaying nociceptive inputs to supraspinal structures. In patients with dementia, the sensory–discriminative aspect of pain, which is referred to as the lateral nociceptive pathway, may be preserved.^^12^^ However, in AD the cognitive–evaluative and emotional–affective dimensions of pain, represented by the medial nociceptive pathway, may be affected due to neurodegeneration within the limbic structures, hippocampus, and prefrontal cortex.^^18^^ Collectively, evidence suggesting elevation in pain tolerance exhibited by patients with AD has made it difficult to understand whether decreased pain complaints are related to altered pain processing or the deteriorating ability of these patients to communicate which hinders their capacity to report pain.^^37^^ Therefore, a better understanding of the pathophysiological mechanisms underlying alterations in sensory transmission is essential for improving the clinical management of pain in patients with AD. In this study, we assessed nociceptive behaviour and explored AD-related pathology in the spinal cord of a double-mutant transgenic TASTPM mouse model of AD that carries mutant versions of the APP (APPswe) and presenilin-1 (PS1.M146V) associated with familial forms of AD.^^16^^

## 2. Methods

### 2.1. Animals

Experiments were performed on 4- to 7-month-old adult male and female heterozygous double-mutant TASTPM transgenic mice. These mice were generated using TAS10 transgenic mice expressing the Swedish mutant human amyloid precursor protein (APP) (695-aa isoform) under the control of the murine Thy-1 promoter and transgenic mice overexpressing presenilin-1 M146V mutation (TPM) driven through the murine Thy-1 promoter.^^32,16^^ Briefly, TAS10 (Thy-1.APPswe) mice were generated and backcrossed onto a pure C57BL/6 background before being crossed with TPM (Thy-1.PSEN-1.M146V) mice to produce heterozygous double-mutant TASTPM mice (GlaxoSmithKline). Age- and gender-matched C57BL/6J obtained from Charles River Laboratories were used as controls. All animals were housed in the Biological Services Unit, King's College London; maintained in 12 hours day/night cycle with access to food and water ad libitum; and were allowed acclimatisation for 7 days before behavioural experiments. All experiments were performed in accordance with United Kingdom Home Office Regulations (Animal Scientific Procedures Act, 1986).

### 2.2. Novel object recognition test

Animals were placed in the neutral test arena, circular open maze (60 × 50 cm) obtained from Ugo Basile (Varese, Italy), and allowed to freely explore it 3 times (10 minutes each) to habituate to both the arena and experimenter before the beginning of the experiment. After each exploration, animals were removed from the arena, which was then cleaned with 70% ethanol. After the habituation period, 2 identical objects (2 × black cube, F) were placed inside the arena, at equal distance from the walls. Mice were placed in the arena at the furthest distance from the objects and allowed to freely explore the objects for 10 minutes. Subsequently, following a retention period of 24 hours, mice were placed back into the arena with one of the familiar objects (F) replaced by one novel object (white sphere, N) in the same location with N being different in colour, shape, and texture. The mice were then allowed to freely explore both objects for 10 minutes. The objects and the arena were thoroughly cleaned with 70% ethanol between each animal to avoid any odour recognition. The mouse was considered to be exploring the object when its head was facing the object at a distance of 2 cm or less. Mice were individually observed and the time taken to explore each object was recorded to the nearest 0.01 seconds. The time difference in exploring each object was calculated:
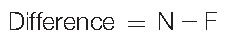


### 2.3. Locomotor function (Roto-Rod)

Locomotor function was assessed using an accelerating Roto-Rod (Series 8, IITC, Los Angeles, CA) with rubber drums set to accelerate from 4 to 40 rpm over a period of 300 seconds. Any mice remaining on the apparatus after 300 seconds were removed and their time was recorded as 300 seconds. Three separate trials were performed for each animal, separated by at least 15 minutes.

### 2.4. Mechanical thresholds: von Frey filaments

Static mechanical withdrawal thresholds were assessed by applying calibrated von Frey monofilaments (0.007-1.00*g*) to the plantar surface of the hind paw. The 50% paw withdrawal threshold (PWT) was determined by increasing or decreasing stimulus intensity and estimated using the Dixon “up–down” method.^^6^^ Unrestrained mice were placed individually and acclimatised up to 60 minutes, prior testing, in acrylic cubicles (8 × 5 × 10 cm) on a wire mesh grid, providing access to the underside of the hind paw. Monofilaments were applied perpendicular to the plantar surface of the selected hind paw and then held in this position with enough force to cause a slight bend in the filament for approximately 3 seconds or until an abrupt withdrawal of the hind paw from the stimulus, the latter defining a positive response. Each test started with the application of 0.07*g* filament and each hind paw was assessed alternately with approximately 30-second gap between each application. Stimulus intensity was increased in a sequence until a positive response was achieved or the maximum strength stimulus of 1.00*g* filament failed to induce a positive response, to avoid tissue damage. If the mouse withdrew its hind paw on application of a filament, the next lower force filament in the sequence was applied and vice versa until there was a change in response from the mouse. Following a positive response, 4 successive filaments were assessed according to the “up–down” sequence, with no filament applied more than 3 times, to prevent sensitization. A 50% PWT (g) was determined using the “up–down” procedure.^^11^^

### 2.5. Thermal thresholds: hot-plate test

Response to noxious heat stimulation of the paws and tail was assessed using the hot plate. Mice were placed on the hot-plate device set at temperature ∼52.5°C (±0.2°C). The latency to respond (licking of hind paw or flicking of hind paw or jumping) was recorded to the nearest 0.01 seconds to obtain the withdrawal response latency. A cutoff time of 45 seconds was used at which point the animals were removed to avoid tissue injury.

### 2.6. Thermal thresholds: Hargreaves test

Thermal nociceptive thresholds of the hind paw were determined by the Hargreaves method using the plantar test (37370; Ugo Basile).^^14^^ Unrestrained mice were acclimatised in acrylic cubicle (8 × 5 × 10 cm) on a uniform glass surface up to 60 minutes before testing. An infrared light source was directed onto the plantar surface of the hind paw, and the paw withdrawal latency was automatically measured in seconds. Three responses for each hind paw were recorded on each testing occasion with at least 1-minute between stimuli. To avoid tissue injury, the maximum stimulus latency period permitted was 20 seconds.

### 2.7. Carrageenan model of inflammatory pain

Carrageenan obtained from Sigma-Aldrich (Gillingham, United Kingdom) was dissolved in sterile saline (0.9% NaCl) at 1% and injected onto the intraplantar surface of the right hind paw of 6- to 7-month-old TASTPM mice and age- and gender-matched C57BL/6J (controls). Mechanical (von Frey filaments) and thermal (Hargreaves test) nociceptive thresholds were recorded before and at intervals of 3 hours and 24 hours after carrageenan injection.

### 2.8. Naloxone administration

Naloxone hydrochloride (1 mg/kg) was obtained from Sigma-Aldrich and dissolved in sterile saline. Either naloxone or saline (vehicle) was administrated intraperitoneally in TASTPM and wild-type (WT) controls. Baseline paw withdrawal latencies were recorded before naloxone administration, and on the day of testing the effect of the drug administrated was monitored over a period of 3 hours, with hot-plate tests performed at 30 minutes, 90 minutes, and 180 minutes after administration.

### 2.9. Immunohistochemistry

Naive WT (6-7 months old) and TASTPM (6-7 months and 12 months old) mice were terminally anaesthetised with an overdose of sodium pentobarbital (∼150 mg/kg body weight, Euthatal, Merial Animal Health, Woking, United Kingdom) and perfused transcardially with heparinised (1 U/mL) sterile saline (0.9% NaCl) followed by 4% paraformaldehyde fixative solution containing 1.5% picric acid in phosphate buffer (PB, 0.1M, pH 7.4). Spinal cords (L3-L6) and brains were removed and immersion-fixed in 4% paraformaldehyde fixative solution containing 1.5% picric acid for 24 hours at 4°C. Subsequently, spinal cords and brains were cryoprotected in a solution of 20% sucrose in PB at 4°C for at least 48 hours and subsequently embedded in optimum cutting temperature (OCT, BDH, Leicestershire, United Kingdom) medium and then snap-frozen using liquid nitrogen and stored at −80°C. Transverse spinal cord and coronal brain sections were cut (20 μm thick) using a cryostat and thaw mounted onto Superfrost Plus microscope slides (BDH). Free-floating spinal cord sections were cut at 30-μm thickness and transferred to phosphate-buffered saline (PBS) in 24-well plates. Slide-mounted sections were blocked with 1% (w/v) bovine serum albumin (BSA, Sigma-Aldrich) and 0.2% (w/v) sodium azide (Sigma-Aldrich) in 0.1% Triton X-100 (BDH) in PBS for 1 hour and incubated overnight for single staining with rabbit anti–met-enkephalin (ENK) and mouse anti–β-amyloid 1-16 (6E10) or with a combination of primary antibodies. Double staining was performed using rabbit anti–vesicular glutamate transporter 1 (VGLUT1) with guinea pig anti–vesicular glutamate transporter 2 (VGLUT2); and mouse anti-6E10 in combination with the following primary antibodies: rabbit anti–glial fibrillary acidic protein (GFAP), rabbit anti–ionized calcium binding adaptor molecule 1 (IBA1), rabbit anti–neuronal nuclei (NeuN), or sheep anti–calcitonin gene-related peptide (CGRP). Details of the source and dilution of the primary antibodies are provided in Table 1. Sections were then incubated for 2 hours with the appropriate Alexa Fluor 488- or 546-conjugated antibody (1:500; Molecular Probes, USA). Where spinal cord sections were stained with 6E10, pretreatment with 70% formic acid was performed. All antibody solutions were prepared in PBS with 1% BSA, 0.1% Triton X-100, and 0.2% sodium azide. Where spinal cord sections were stained with VGLUT1 and VGLUT2, 10% BSA was used in antibody solution instead of 1%. Free-floating spinal cord sections were blocked with 1% BSA, 0.1% Triton X-100, and 0.2% sodium azide in PBS and incubated for 72 hours on a shaker at 4°C with the primary antibodies (Table 1): mouse anti-6E10 and rabbit anti–neurokinin 1 receptor (NK1R). Sections were then incubated with the appropriate Alexa Fluor 488- or 546-conjugated antibody (1:500; Molecular Probes, USA) overnight on a shaker at 4°C and then mounted onto microscope slides. All slides were coverslipped with the Vectashield Mounting Medium containing nuclear marker 4′,6-diamidino-2-phenylindole·2HCl (DAPI; Vector Laboratories, Peterborough, United Kingdom), and fluorescent staining was visualized using the Zeiss LSM710 confocal microscope (Zeiss, Cambridge, United Kingdom).

**Table 1.**
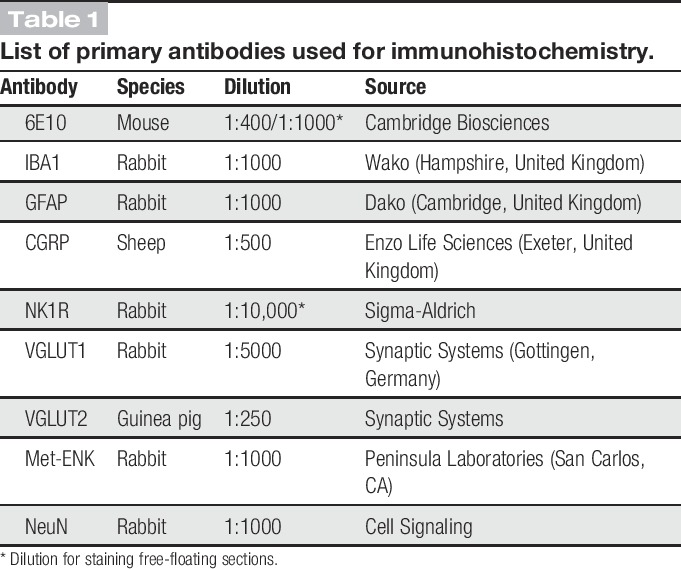
List of primary antibodies used for immunohistochemistry.

### 2.10. Quantitative assessment of fluorescence intensity

Quantitative assessment of VGLUT1, VGLUT2, and met-ENK immunoreactivity was calculated by determining immunofluorescence intensity within 1 × 10^4^ μm^2^ boxes placed onto areas of the lateral, central, and medial dorsal horn laminae I-III and laminae IV-VI (VGLUT1 and VGLUT2 only) using Axiovision LE 4.8 software (Zeiss). Background fluorescence intensity of each tissue section was also determined and subtracted from the values obtained. Three L3-L5 sections (at least 160 μm apart) from each animal were randomly selected from at least 4 animals per experimental group.

### 2.11. Western blots

The lumbar spinal cord (L3-L5) and brain (cerebral cortex) tissue from 6- to 7-month-old WT and TASTPM mice were homogenized on ice in lysis buffer (Tris-HCl, 20 mM pH 7.5, 10 mM NaF, 150 mM NaCl, 1% Nonidet P-40, 1 mM phenylmethylsulfonyl fluoride fluoride, 1 mM Na_3_VO_4_, 10 μg/mL leupeptin and trypsin inhibitor) with complete mini cocktail protease inhibitor (Roche). Tissue lysates were then centrifuged at 13,000 rpm for 20 minutes at 4°C. The protein concentration of the supernatant was determined using the NanoDrop spectrometer. Equal protein concentrations per sample (60 μg protein) were added to Laemmli sample buffer, boiled for 5 minutes, and subjected to 10% to 18% SDS-PAGE. Wet transfer was performed using the Bio-Rad Trans-Blot Cell (Bio-Rad Laboratories, Hertfordshire, United Kingdom) for 1 hour at 4°C, and the membrane was then blocked with 5% non–fat-dried milk in TBS-T (50 mM Tris-HCl, pH 7.6, 150 mM NaCl, 0.1% Tween 20) for 30 minutes at room temperature. The blot was probed with rabbit anti-VGLUT1 (1:1000; Cell Signaling, Hertfordshire, United Kingdom), rabbit anti-VGLUT2 (1:1000; Cell Signaling), and mouse anti-amyloid 1 to 16 (1:1000, 6E10; Cambridge Biosciences, United Kingdom) antibodies. Results were visualised with horseradish peroxidase–coupled anti-mouse or anti-rabbit immunoglobulin (Dako) using enhanced chemiluminescence detection reagents ECL (EMD Millipore, Hertfordshire, United Kingdom), Western blotting detection system according to the manufacturer's instructions, and the immune complex visualized by the BioSpectrum Imaging System. The protein bands were densitometrically analyzed with Quantity One (Bio-Rad Laboratories). Western blot for β-actin (1:1000; Cell Signaling) was performed as control. Data were expressed as protein expression relative to control.

### 2.12. Real-time PCR

Lumbar segments (L3-L5) of the spinal cord were dissected and snap-frozen in liquid nitrogen and subsequently divided into dorsal and ventral horn. Total RNA was then isolated from minced dorsal horn tissue using the RNeasy mini-kit (Qiagen) according to the manufacturer's protocol. The total RNA concentration was determined using the NanoDrop spectrometer. Total RNA (50-100 ng) was used to synthesize first strand cDNA, using Supersensitive III Reverse Transcriptase kit (Thermo Fisher Science) according to the manufacturer's protocol. Expression levels of the following genes were analysed: preproenkephalin (pPENK), proenkephalin (PENK), and 18S rRNA were used reference transcripts. pPENK is the precursor for PENK which contains peptides with 7 amino acid sequences and produces enkephalin peptide.^^10^^ Amplification was performed with a Light Cycler 480 (Roche) using Syber Green I Master (Roche) using the primers: pPENK (TTCAGCAGATCGGAGGAGTTG and AGAAGCGAACGGAGGAGAGAT),^^9^^ PENK (ATGCAGCTACCGCCTGGTT and GTGTGCACGCCAGGAAATT),^^23^^ and 18S rRNA (GCTGGAATTACCGCGGCT and CGGCTACCACATCCAAGGAA).^^9^^ The instrument was programmed as follows: 95°C for 5 minutes and 45 cycles of 3 steps of 10 seconds each including denaturing at 95°C, annealing at 60°C, and primer extending at 72°C. All samples were run as duplicates and the 18S rRNA was used as the housekeeping gene. The relative gene expression levels were calculated according to the 2^–ΔΔCt^ method, where Ct represents the threshold cycle.

### 2.13. Statistical analysis

The data were analysed using SigmaPlot 12.5 (Systat Software, San Jose, CA). The statistical tests performed and the numbers of animals used are displayed in the Results section and within the figure legends. Where data were not normally distributed, the appropriate nonparametric test was applied. All data are presented as mean ± SEM and a probability value less than 0.05 (*P* < 0.05) was considered statistically significant.

## 3. Results

**Figure 1.**
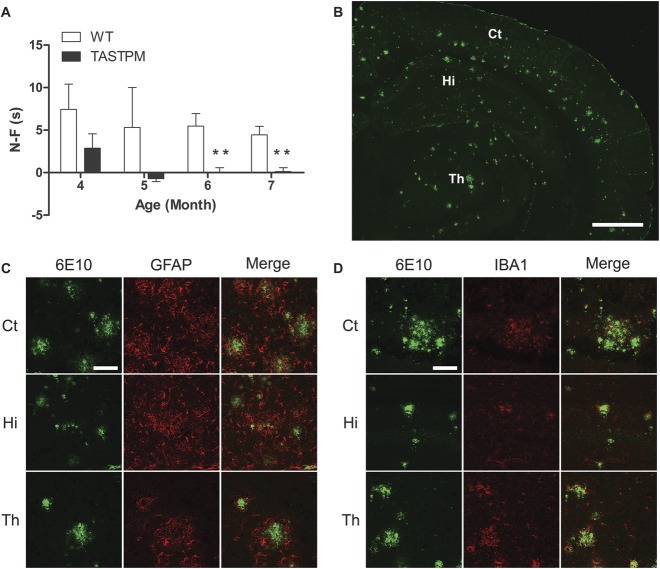
Cognitive and pathological features of the TASTPM mouse model of Alzheimer's disease (AD). TASTPM (9 males and 6 females) mice displayed memory deficits compared with wild-type (WT) mice (5 males and 5 females) at the age of 6 and 7 months but not earlier (A) (**P < 0.01, *t* test or Mann–Whitney Rank Sum test). Data values are expressed as mean ± SEM (n = 10-15 per experimental group). Coronal brain sections from TASTPM mice (male) displayed AD-related pathological hallmark namely amyloid plaques composed of aggregated Aβ (B) accompanied by glial fibrillary acidic protein (GFAP)-positive astrocytes (C) and ionized calcium binding adaptor molecule 1 (IBA1)-labelled microglia (D) forming a barrier around Aβ pathology. The scale bar represents 1 mm (B) and 100 μm (C–D). Ct, cerebral cortex; Hi, hippocampus; Th, thalamus.

**Figure 2.**
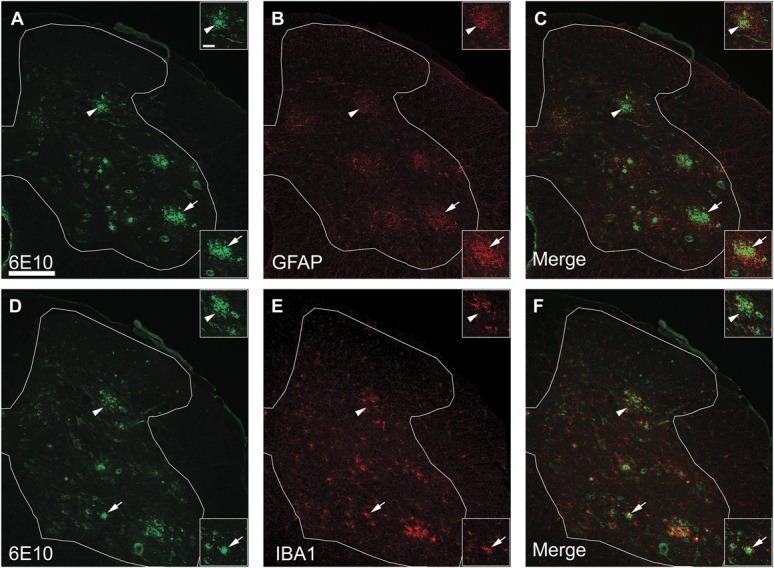
Aβ deposits in the spinal cord of 12-month-old TASTPM mice. Transverse lumbar spinal cord sections from 12-month-old TASTPM mice (male) were stained with antibodies against β-amyloid 1-16 (6E10) (A and D) with either glial fibrillary acidic protein (GFAP) (B) or ionized calcium binding adaptor molecule 1 (IBA1) (E). Merged images are shown for 6E10 with GFAP (C) or IBA1 (F) co-immunostaining in the spinal cord. Pathological amyloid plaques composed of Aβ are evident in the dorsal (arrowheads) and ventral horns (arrows) of the spinal cord (A and D). A high-power magnification (insets) revealed GFAP (A–C) and IBA1 (D–F) immunoreactivity surrounding the 6E10-labelled amyloid plaques in the dorsal and ventral horns. Scale bars: 200 μm (A–F) and 50 μm (insets).

**Figure 3.**
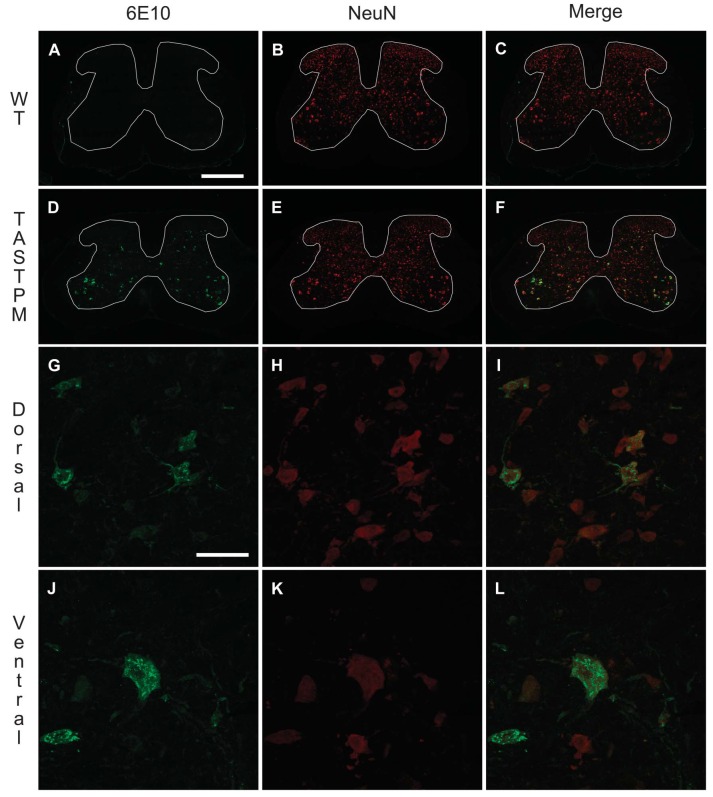
Aβ expression in the spinal cord. Transverse lumbar spinal cord sections from 6- to 7-month-old TASTPM and wild-type (WT) male mice were stained with antibodies against β-amyloid 1-16 (6E10) (A, D, G, and J) and neuronal nuclei marker (NeuN) (B, E, H, and K). Merged images are shown for 6E10 and NeuN co-immunostaining in the spinal cord of WT (C) and TASTPM (F) mice as well as TASTPM dorsal horn (I) and ventral horn (L). 6E10 staining was only detected in the spinal cord of the transgenic TASTPM mice and was distributed throughout the grey matter. A high-power magnification revealed colocalisation of 6E10 and NeuN in the dorsal horn laminae III-IV and motor neurons in the ventral horn of the transgenic TASTPM mice spinal cord. Scale bars: 500 μm (A–F) and 50 μm (G–L).

As expected, TASTPM mice displayed age-dependant memory deficits in the novel object recognition test, as they spent significantly less time exploring the novel object compared with WT mice at the age of 6 and 7 months, but not at the age of 4 and 5 months (Fig. 1A). Obvious extracellular amyloid plaques, composed of aggregated Aβ, were observed in the brain of 6-month-old TASTPM mice. These plaques were located specifically in the cortex and hippocampus as well as in the thalamus (Fig. 1B). Amyloid plaques in the brain were surrounded by barriers formed by astrocytes (GFAP, Fig. 1C) and microglia (IBA1, Fig. 1D). Previous studies have provided evidence for dystrophic neurites.^^15^^ In the spinal cord, amyloid deposits were detected in 12-month-old TASTPM mice and these deposits were accompanied by barriers formed by astrocytes (GFAP, Fig. 2A–C) and microglia (IBA1, Fig. 2D–F). However, no plaque pathology was observed in the spinal cord of 6-month-old TASTPM mice (Fig. 3).

**Figure 4.**
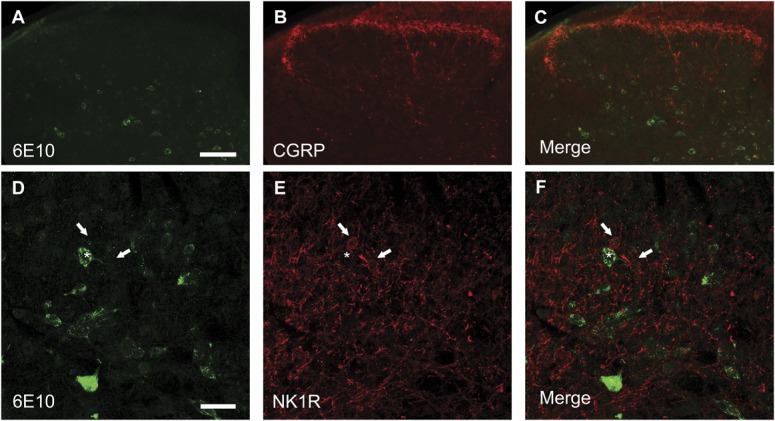
Aβ absent in primary afferent terminals and NK1 receptor–positive neurons. Transverse spinal cord sections from 6- to 7-month old TASTPM mice (male) were stained with antibodies against β-amyloid 1-16 (6E10) (A and D), calcitonin gene-related peptide (CGRP) (B) and neurokinin 1 receptor (NK1R) (E). Merged images are shown for 6E10 costained with CGRP (C) and NK1R (F). β-amyloid 1-16 was absent in primary afferent CGRP-immunopositive terminals and was also not present in NK1R-positive neurons (arrows) in the dorsal horn lamina III of the TASTPM spinal cord. Scale bars: 100 μm (A–C) and 30 μm (D–F).

**Figure 5.**
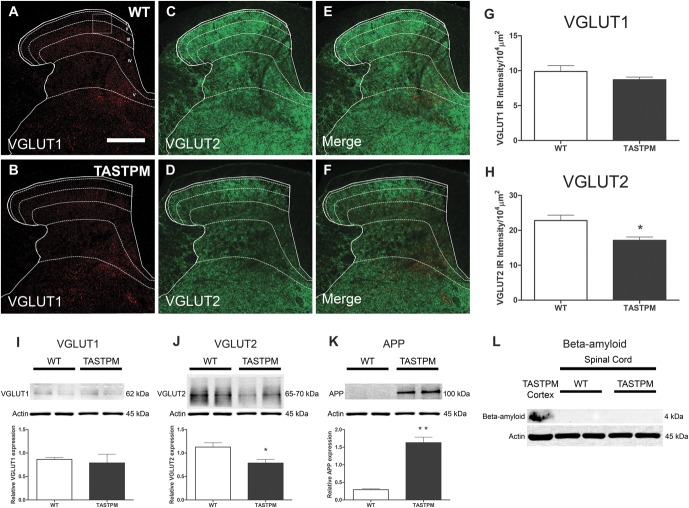
Aβ and VGLUTs expression in the spinal cord. Transverse spinal cord sections from 6- to 7-month-old TASTPM and wild-type (WT) mice (male) were stained with antibodies against vesicular glutamate transporter 1 (VGLUT1) (A and D) and vesicular glutamate transporter 2 (VGLUT2) (B and E). Merged images are shown for VGLUT1 costaining with VGLUT2 (C and F). The distribution of VGLUT1 in TASTPM and WT spinal cord was mainly in the deeper dorsal horn laminae (III-VI) (A and D), while VGLUT2 was found throughout the grey matter but with less prominence in the medial part of the TASTPM deep dorsal horn (B and E). Quantitative analysis of VGLUT1 and VGLUT2 immunostaining intensity in the dorsal horn revealed significantly reduced VGLUT2 intensity in the dorsal horn of the TASTPM mice compared with WT controls (*P < 0.05, *t* test, H) but no difference in VGLUT1 intensity (*P* > 0.05, *t* test, G). Data values are expressed as mean ± SEM (n = 4 (2 males and 2 females) per experimental group). Scale bars: 200 μm (A–F). Quantitative levels of VGLUTs, amyloid precursor protein (APP), and β-amyloid (Aβ) determined by Western blot revealed significantly higher expression of APP and reduced levels of VGLUT2 in the lumbar (L3-L5) spinal cord of TASTPM mice compared with WT controls (*P < 0.05, **P < 0.01, *t* test) relative to β-actin (loading control) (I–K). Aβ peptide was only detected in the TASTPM cerebral cortex, however, absent in both WT and TASTPM spinal cords (L). Data values are expressed as mean ± SEM (n = 3 [1 male and 2 females] per experimental group).

**Figure 6.**
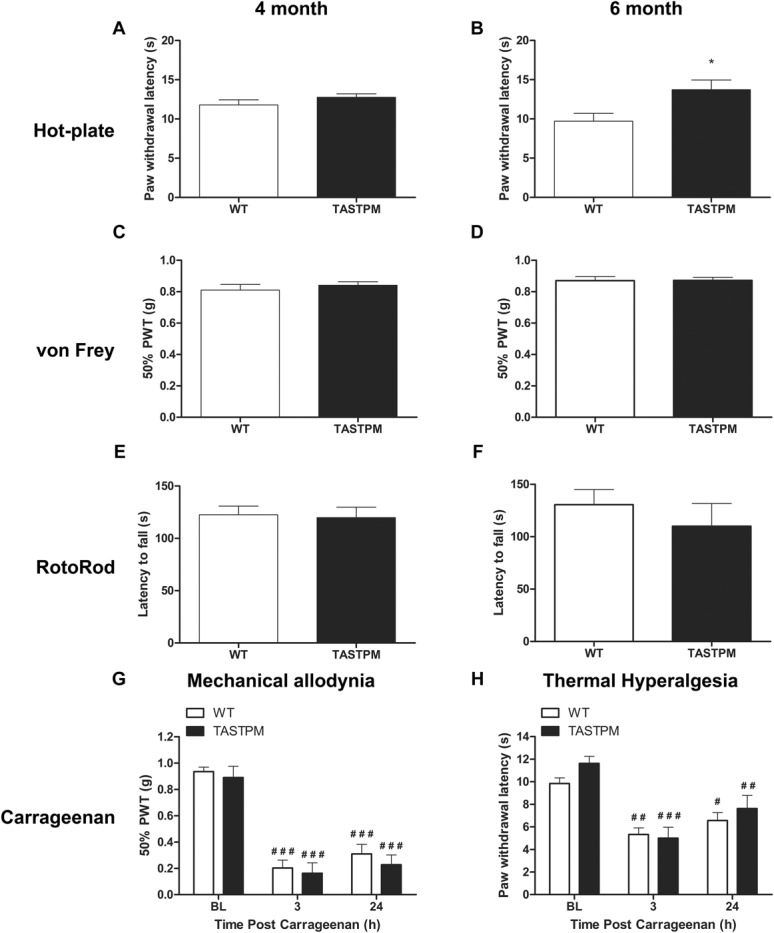
Nociceptive thresholds and motor coordination. TASTPM mice (9 males and 7 females) displayed comparable nociceptive thresholds to wild-type (WT) (5 males and 5 females) mice in response to noxious thermal stimulation at the age of 4 months (A); however, an increased paw withdrawal latency compared with WT mice at the age of 6 months (B) (*P < 0.05, Mann-Whitney Rank Sum test). TASTPM mice exhibited responses comparable with WT mice in response to noxious mechanical stimulation (C and D). No motor deficit in the TASTPM mice at the age of 4 and 6 months, as no significant difference in the latency to fall from an accelerating RotoRod was observed when compared with WT controls (E and F). Data are expressed as mean ± SEM (n = 10-16 per experimental group). Mechanical thresholds (G) and thermal withdrawal latencies (H) of the ipsilateral hind paw in response to intraplantar carrageenan (1%) injection in 6- to 7-month-old TASTPM and WT mice. Both TASTPM and WT mice developed mechanical allodynia and thermal hyperalgesia 3 hours postcarrageenan, which persisted at 24 hours. At no point were there any significant differences in behavioural responses of the TASTPM (2 males and 3 females) compared with WT controls (1 male and 3 females). Data are presented as mean ± SEM (n = 4-5 per experimental group); 2-way repeated-measures analysis of variance followed by post hoc Tukey multiple comparison test (#*P* < 0.05, ##*P* < 0.01, and ###*P* < 0.001 compared with respective baseline).

In the spinal cords of 6-month-old TASTPM mice, but not WT mice, we observed APP/Aβ expression in the grey matter (Fig. 3A and D). APP/Aβ expression was mainly neuronal, as it was observed in NeuN-positive cells in the TASTPM mice (Fig. 3D–F) while absent in WT mice (Fig. 3A–C). APP/Aβ was evidently expressed in neurons in deep dorsal horn laminae (Fig. 3G–I) and in motor neurons in the ventral horn (Fig. 3J–L). In the dorsal horn, APP/Aβ was not coexpressed with the primary afferent marker CGRP in laminae I and II (Fig. 4A–C). Similarly, APP/Aβ did not colocalise with NK1 receptor in neurons which are presumably a subpopulation of projection neurons in lamina III (Fig. 4D–F). To evaluate possible changes in excitatory function in TASTPM mice spinal cord, we assessed the expression of vesicular glutamate transporters VGLUT1 and VGLUT2. As previously reported,^^4^^ the distribution of VGLUT1 profiles in WT spinal cords was mainly in the deeper dorsal horn laminae (III-V) (Fig. 5A), and we observed that this was not altered in the spinal cords of TASTPM mice (Fig. 5B). As reported, VGLUT2 immunoreactivity was found throughout the grey matter in WT spinal cords (Fig. 5C), but we observed that VGLUT2 immunostaining was less prominent in the medial part of the deep dorsal horn in TASTPM spinal cords (Fig. 5D). Quantitative analysis of VGLUT1 and VGLUT2 immunostaining intensity in the dorsal horn revealed significantly reduced VGLUT2 staining intensity in the dorsal horn of the TASTPM mice compared with WT mice, while no difference in the expression of VGLUT1 was observed (Fig. 5G and H). Furthermore, Western blot analysis of VGLUT1 and VGLUT2 proteins in the lumbar spinal cord showed significantly lower expression of VGLUT2, but not VGLUT1 in TASTPM mouse tissue compared with WT mice controls (Fig. 5I and J). Predictably, the expression of APP was present only in the TASTPM spinal cords (Fig. 5K); however, Aβ peptide was undetectable in the spinal cord but observed in the cortex of the transgenic TASTPM mice (Fig. 5L), where amyloid plaques were detected (Fig. 1B). As in 6-month-old TASTPM mice the presence of APP in the spinal cord is associated with a significant decrease in expression of VGLUT2, these data suggest possible alterations of the excitatory function. Many deep dorsal horn neurons respond to noxious input and also send their dorsal dendrites superficially into laminae I-III where they receive primary afferent input from unmyelinated sensory fibres which respond to noxious stimuli in the periphery.^^46^^ Thus, although we observed no significant APP/Aβ expression in superficial laminae, we evaluated whether TASTPM mice display sensory changes compared with WT mice. TASTPM mice displayed increased response latency to thermal stimulation compared with WT mice at the age of 6 months, but not at the age of 4 months (Fig. 6A and B). However, hind PWTs to mechanical stimulation in the TASTPM mice were comparable with WT mice at the age of 4 and 6 months (Fig. 6C and D); and motor coordination was no different from WT mice in any age group (Fig. 6E and F). In the carrageenan model of inflammatory pain, we observed comparable development of mechanical allodynia (Fig. 6G) and thermal hyperalgesia (Fig. 6H) in the ipsilateral hind paw of 6- to 7-month-old TASTPM and WT mice at 3 hours after injection which was maintained at 24 hours. Therefore, mice displaying amyloid plaques in the brain and cognitive deficits, which are both indicative of development of AD-like pathology in the brain, also display accumulation of APP/Aβ in spinal cord neurons and reduced sensitivity to acute noxious heat stimuli applied to the hind paw in the periphery. However, TASTPM mice responded normally to mechanical and thermal noxious stimuli in a model of peripheral inflammatory pain.

Notwithstanding the importance and relevance of the higher structures in the brain in the perception and modulation of pain, we focused this study on the dorsal horn of the spinal cord which is the first relay station of noxious heat stimuli from the periphery. In particular, we tested the hypothesis that an increased inhibitory tone may underlie reduced thermal sensitivity in TASTPM mice. Specifically, we examined the expression of the enkephalins, in TASTPM mice which are reported to have high levels of opioid peptides in the CSF similarly to patients with AD.^^29,33^^

**Figure 7.**
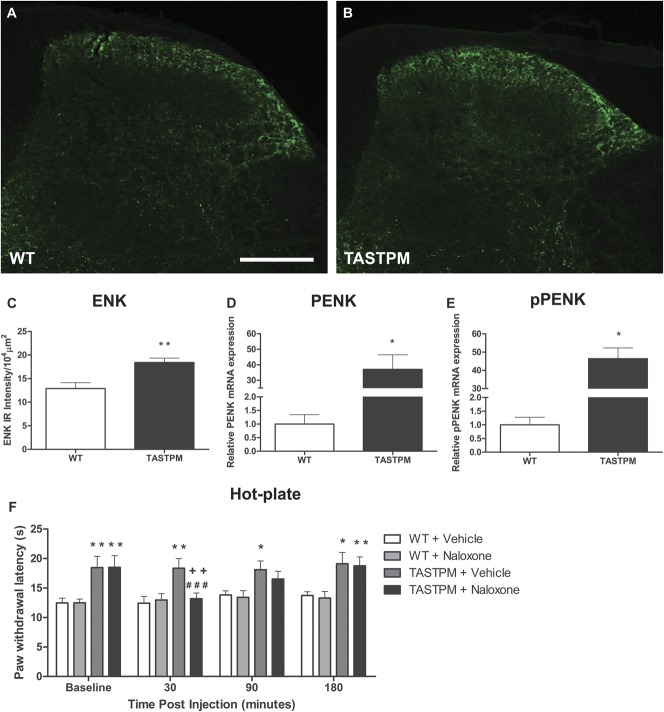
Expression of enkephalins in the spinal cord and effect of naloxone on thermal thresholds. Transverse lumbar spinal cord sections from 6- to 7-month-old TASTPM (5 males and 5 females) and wild-type (WT) (4 males and 4 females) mice were stained with met-enkephalin (ENK) (A and B). Quantitative analysis of met-ENK immunostaining in the dorsal horn revealed significantly higher met-ENK intensity in the dorsal horn of TASTPM compared with WT controls (**P < 0.01, Mann-Whitney Rank Sum test, C). Values are expressed as mean ± SEM (n = 8-10 per experimental group) and scale bar represents 200 μm. In addition, RT-PCR displayed significantly higher mRNA expression of preproenkephalin (pPENK, D) and proenkephalin (PENK, E) in 6- to 7-month-old TASTPM (2 males and 2 females) dorsal horn (L3-L5) compared age-matched WT (4 females) controls (*P < 0.05, Mann-Whitney Rank Sum test). Data are expressed as mean ± SEM (n = 4 per experimental group). Administration of naloxone (1 mg/kg intraperitoneally), an opioid antagonist, reduced paw withdrawal latency in 6- to 7-month-old TASTPM mice (4 males and 5 females) compared with baseline (###*P* < 0.001) and TASTPM saline (4 males and 5 females) control (++*P* < 0.01) after 30 minutes. However, there was no effect of naloxone observed in WT mice (5 males and 5 females) compared with their respective baseline and WT saline (5 males and 5 females) control. TASTPM mice exhibited significantly higher paw withdrawal latency compared with WT mice injected with saline at baseline and recovered back to baseline levels and significantly higher 3 hours after naloxone administration (*P < 0.05, **P < 0.01) using 2-way repeated-measures analysis of variance followed by Tukey multiple comparison test (F). Data are presented as mean ± SEM (n = 9-10 per experimental group).

We observed a significant increase of enkephalin immunoreactivity in laminae I and II of the dorsal horn of 6-month-old TASTPM mice compared with WT mice (Fig. 7A–C). Furthermore, preproenkephalin (pPENK) and proenkephalin (PENK) mRNA expression in TASTPM mice dorsal horn tissue extracts was significantly higher than that in control tissue (Fig. 7D and E). The preproenkephalins and proenkephalins are processed to generate the enkephalin peptides.^^10^^ These data suggest an increased opioidergic tone in TASTPM mice due to increased production and release of enkephalins in the spinal cord. To test this possibility, we administered the opioid receptor antagonist naloxone and observed a significant reduction of TASTPM mice thermal thresholds at 30 minutes from injection compared with both baseline and saline control values (Fig. 7F), whereas naloxone administration did not alter thresholds in WT mice. Altogether, these data suggest that there is an age-dependant alteration in thermal sensitivity which coincides with the onset of cognitive deficits. Intracellular APP/Aβ expression was detected only in the spinal cords of TASTPM mice and was accompanied by alterations in both the glutamatergic and opioidergic systems in the spinal cord.

## 4. Discussion

In this study, we provide evidence for intraneuronal APP/Aβ accumulation in the spinal cords of TASTPM mice, in both dorsal and ventral grey matters, thereby highlighting the presence of early AD-like pathology in the spinal cord of 6-month-old TASTPM mice. Further analysis revealed a decrease of glutamatergic interneuron marker VGLUT2 in the dorsal horn of TASTPM mice compared with WT mice. Glutamate is present in primary afferent terminals, projection neurons, and excitatory interneurons in the spinal cord where it plays pronociceptive roles.^^45^^ In nociceptive behavioural studies, the transgenic mice exhibited an age-dependant decline in sensitivity to thermal stimulation which could be attributed to a reduced glutamatergic excitation and/or increased inhibition in the spinal cord. Indeed, we found higher levels of pre–pro and proenkephalin mRNA and enkephalin peptide expression in the dorsal horn of TASTPM mice suggesting that an increased synthesis and release of enkephalins in the spinal cord could result in elevated inhibitory tone. This likely possibility is strengthened by our finding of the reversal of thermal hyposensitivity following administration of the opioid antagonist naloxone in TASTPM mice.

Cognitive deficits and amyloid plaques are among the major clinical and pathological hallmarks of AD in the brain, respectively. As expected, TASTPM mice display an age-dependant cognitive impairment in the object recognition test which becomes apparent at the age of 6 months. In addition, extracellular amyloid plaques accompanied by gliosis are present in the cerebral cortex and hippocampus as well as the thalamus, a key relay station in nociceptive signalling pathways.^^15^^ Collectively, these findings are consistent with previous reports indicating that the TASTPM mouse model recapitulates some of the key features of AD clinically and pathologically.^^15,16^^

The novelty of this study is in providing evidence for intraneuronal accumulation of APP/Aβ in both the dorsal (lamina III and deeper) and ventral horns of the lumbar spinal cords of TASTPM mice, while previous studies have primarily focused on the brain. Previous studies have reported Aβ pathology in the 5XFAD and Tg2576 transgenic mouse models of AD, but have mainly focused on the dorsal column or the ventral horn and suggested an association between intraneuronal Aβ and motor impairment.^^21,39,47^^ Differently, in this study despite the accumulation of APP/Aβ in ventral horns, no gross motor deficits were detected in the TASTPM mice using the accelerating RotoRod test. However, this may have been due to the fact that the RotoRod may not have been a sensitive enough test to detect subtle motor deficits.^^3^^ Furthermore, our study highlights the presence of APP/Aβ accumulation in both dorsal and ventral horn neurons and assesses the impact on sensory and motor behaviour in the transgenic TASTPM mice compared with WT mice controls.

Intraneuronal Aβ is associated with synaptic dysfunction and early cognitive impairments before the development of the AD pathological hallmark in the form of extracellular plaques.^^30,19^^ The intracellular pool of Aβ may act as a source for the formation of extracellular plaques and the intracellular Aβ intensity decreases as the AD core pathology develops.^^27^^ Further evidence illustrates that intracellular Aβ accumulates in multivesicular bodies of neurons within the presynaptic and postsynaptic compartments and a study in 3xTg model of AD revealed synaptic dysfunction and long-term potentiating (LTP) deficit before the presence of amyloid plaques.^^42,30^^ Therefore, we speculate that intraneuronal accumulation of APP/Aβ in the spinal cord of 6-month-old TASTPM mice is an early-stage AD-like pathological feature which has the potential to induce synaptic dysfunction and contribute towards the development of amyloid plaques, as observed in the spinal cords of 12-month-old TASTPM mice, with progression of the disease.

APP/Aβ were not found in primary afferent nociceptive terminals in the superficial (laminae I-II) nor in the lamina I-III projection neurons. Following activation by noxious stimuli in the periphery, a subpopulation of primary afferent fibres release CGRP and substance P (SP) alongside glutamate from their central terminals in the dorsal horn and mediate nociceptive transmission through activation of CGRP and NK1 receptors in projection neurons. Ablation of NK1-R resulted in a significant attenuation of responses to highly noxious stimuli (capsaicin) and mechanical hyperalgesia.^^24^^ Whilst CGRP contributes in nociceptive transmission directly and indirectly by potentiating the actions of SP through: promoting release, inhibition of degradation, and regulating the expression of NK1-R.^^26^^ Therefore, the lack of APP/Aβ immunoreactivity in an NK1-R–labelled subpopulation of presumed projection neurons implies that APP/Aβ may not directly affect the neurons relaying nociceptive information to the supraspinal structures. Notably, recent evidence suggests that there may be a species difference between the mouse and rat, as many of the large lamina III anterolateral tract neurons in the mouse do not show NK1 receptor immunoreactivity.^^5^^ We therefore cannot rule out the possibility that lamina III anterolateral tract neurons that lack the NK1 receptor may express APP/Aβ. However, APP/Aβ immunoreactivity was present in deep dorsal horn neurons where we detected a reduction in VGLUT2 expression in the lumbar segment of the spinal cord and especially in the dorsal horn laminae IV-V. This finding may suggest that the presence of APP/Aβ is associated with glutamatergic dysfunction. Many lamina IV neurons send their dorsal dendrites superficially into laminae I-III and can receive primary afferent input from unmyelinated sensory fibres which respond to noxious stimuli in the periphery.^^46^^ Thus, the presence of APP/Aβ in the dorsal horn of the spinal cord may indicate an alteration of glutamatergic function that, in turn, could reduce nociceptive transmission and/or excitatory modulation.

In TASTPM mice, thresholds to noxious mechanical stimuli, which evoke a spinal-mediated withdrawal reflex, were similar to that of the WT mice controls. Similarly, after hind paw inflammation, mechanical and thermal hypersensitivity developed with no difference between WT and TASTPM mice. However, there was an age-dependant decrease in thresholds to noxious stimulation in the hot-plate test, which also involves supraspinal integration of nociceptive signals.^^13^^ Consistent with these observations, reduced sensitivity to noxious thermal stimuli has been observed to occur before any changes in mechanical nociceptive thresholds in the CRND8 model of AD^^41^^ and by us in the tail immersion test in TASTPM mice.^^8^^ Therefore, there may be a correlation between impaired nociceptive responses and an increase in APP/Aβ expression in the spinal cord along with cognitive impairment. Intriguingly, a longitudinal study found that patients with AD exhibit reduced pain intensity and affect when compared with individuals without dementia; thereby, supporting the possibility that the pain experience is altered in AD^^35^^ and that the spinal expression of APP/Aβ may be a contributing factor.

To explore the underlying reason for the alteration of TASTPM thermal nociceptive thresholds, the impact of opioid levels on nociceptive behavioural responses was assessed. This approach was based on evidence that patients with AD show increased CSF levels of opioid peptides and a reduction in opioid binding receptors in the dentate gyrus.^^20,29,33^^ In this study, the opioid receptor antagonist, naloxone, induced an increase in sensitivity to thermal stimulation suggesting that increased opioidergic tone in the transgenic mice is responsible for the altered thermal thresholds. Furthermore, histological and quantitative mRNA expression analysis revealed increased expression of enkephalins in the dorsal horn of the spinal cords of TASTPM mice compared with WT mice. In a transgenic mouse model that overexpresses the human APP (hAPP), increased levels of enkephalins have been found in the hippocampus and entorhinal cortex which are associated with neuronal and cognitive impairments.^^28^^ In addition in an APP/PS transgenic mouse model of AD, evidence reported in knockdown or antagonism of the delta opioid receptor has revealed reductions in Aβ accumulation, plaque formation and associated gliosis, and behavioural deficits, a result that supports the role of opioid peptides in Aβ generation.^^44^^

Altogether, these data in a transgenic mouse model of AD show an age-dependant decline in sensitivity to acute noxious thermal stimulation in a model that includes both spinal- and supraspinal-mediated components. Thermal hyposensitivity coincides with cognitive deficits and pathological amyloid plaques in the brain as well as histopathological Aβ plaques and intraneuronal accumulation of APP/Aβ in key regions involved in nociceptive processing, namely the thalamus and spinal cord, respectively.

The accumulation of intraneuronal APP/Aβ in the spinal cord was associated with an alteration of possible excitatory neuronal markers, especially in the deeper laminae coupled with an increased expression of endogenous opioid in superficial laminae of the dorsal horn of the spinal cord. An increased inhibitory tone mediated by endogenous opioid is likely to contribute significantly to thermal hypoalgesia in AD transgenic mice.

Taken together, the findings of this study could have important clinical implications for the care of patients with AD who have deteriorating cognitive function along with reduced sensitivity to pain, as altered thermal perception could be considered in assessing clinical risk and may be associated with an increased risk of injury during daily routine activities.

## Conflict of interest statement

Y. Aman was supported by the Medical Research Council PhD Studentship. The remaining authors have no conflicts of interest to declare.
